# C-Reactive Protein for Early Diagnosis and Severity Monitoring in Melioidosis: A Systematic Review and Meta-Analysis

**DOI:** 10.3390/life15091360

**Published:** 2025-08-27

**Authors:** Atthaphong Phongphithakchai, Moragot Chatatikun, Jitabanjong Tangpong, Sa-ngob Laklaeng, Jason C. Huang, Pakpoom Wongyikul, Phichayut Phinyo, Jongkonnee Thanasai, Supphachoke Khemla, Chaimongkhon Chanthot, Anchalee Chittamma, Wiyada Kwanhian Klangbud

**Affiliations:** 1 Nephrology Unit, Division of Internal Medicine, Faculty of Medicine, Prince of Songkla University, Songkhla 90110, Thailand; atthaphong.p@psu.ac.th; 2 School of Allied Health Sciences, Walailak University, Nakhon Si Thammarat 80160, Thailand; rjitbanj@wu.ac.th (J.T.); sumoun2528@gmail.com (S.-n.L.); 3 Research Excellence Center for Innovation and Health Products (RECIHP), Walailak University, Nakhon Si Thammarat 80160, Thailand; 4 Department of Biotechnology and Laboratory Science in Medicine, National Yang Ming Chiao Tung University, Taipei 112304, Taiwan; jasonhuang@nycu.edu.tw; 5 Center for Clinical Epidemiology and Clinical Statistics, Faculty of Medicine, Chiang Mai University, Chiang Mai 50200, Thailand; aumkidify@gmail.com (P.W.); phichayutphinyo@gmail.com (P.P.); 6 Biomedical Informatics and Clinical Epidemiology Department, Faculty of Medicine, Chiang Mai University, Chiang Mai 50200, Thailand; 7 Faculty of Medicine, Mahasarakham University, Mahasarakham 44000, Thailand; jongkonnee@msu.ac.th; 8 Division of Infectious Diseases, Department of Internal Medicine, Nakhon Phanom Hospital, Nakhon Phanom 48000, Thailand; sup.mednkp@gmail.com; 9 Project for the Establishment of the Faculty of Medicine, Nakhon Phanom University, Nakhon Phanom 48000, Thailand; chaimongkhon251269@gmail.com; 10 Department of Pathology, Faculty of Medicine Ramathibodi Hospital, Mahidol University, Bangkok 10400, Thailand; anchalee.chi@mahidol.ac.th; 11 Medical Technology Program, Faculty of Science, Nakhon Phanom University, Nakhon Phanom 48000, Thailand

**Keywords:** melioidosis, C-reactive protein, CRP, inflammatory biomarker, disease severity, meta-analysis

## Abstract

**Background**: Melioidosis, caused by *Burkholderia pseudomallei*, is a serious infectious disease in Southeast Asia and northern Australia. **Methods**: We systematically reviewed observational studies measuring C-reactive protein (CRP) in laboratory-confirmed melioidosis for diagnosis, severity assessment, or outcome evaluation. PubMed, Embase, and Scopus were searched up to May 2025. Data were pooled using a random-effects model; heterogeneity was quantified (*I*^2^). **Results**: Seven studies (*n* = 451) were included. The pooled mean CRP level in melioidosis was 74.37 mg/L (95% Confidence Interval [CI], 32.76–168.83; *I*^2^ = 99.1%), considerably higher than healthy reference values (<10 mg/L). **Conclusions**: CRP is consistently raised in melioidosis and may aid in early diagnosis and severity monitoring, although high heterogeneity limits the precision of pooled estimates. Integration of CRP into multimodal prediction tools, rather than use in isolation, is recommended. Further prospective studies should define optimal diagnostic thresholds.

## 1. Introduction

Melioidosis is a potentially life-threatening infectious disease caused by the Gram-negative, facultative intracellular bacterium *Burkholderia pseudomallei*. The organism is commonly found in soil and surface water in endemic regions such as northern Australia and Southeast Asia [1,2]. Infection typically occurs through percutaneous inoculation, inhalation, or ingestion. *B. pseudomallei* is notable for its intrinsic resistance to multiple antibiotics, its capacity for intracellular persistence, and its potential to cause latent or relapsing infections [3]. Clinically, melioidosis presents a wide spectrum of manifestations, ranging from localized abscesses and pneumonia to septicemia and multiorgan failure. The disease has a predilection for individuals with underlying comorbidities such as diabetes mellitus, chronic kidney disease, or hazardous alcohol use. In severe cases, especially in resource-limited settings, the case fatality rate may approach 40% [4,5]. Early diagnosis and accurate assessment of disease severity are critical for guiding clinical management and improving survival outcomes. However, culture-based confirmation of *B. pseudomallei* remains time-consuming and requires specialized laboratory capacity, posing challenges for timely diagnosis and risk stratification in endemic areas [6].

C-reactive protein (CRP) is an acute-phase reactant synthesized by hepatocytes in response to inflammatory cytokines, particularly interleukin-6 (IL-6). It is widely utilized in clinical practice as a non-specific biomarker of inflammation and infection. In bacterial infections, elevated CRP levels are associated with disease severity, and CRP is frequently used to assist in the diagnosis of sepsis and pneumonia [7]. Due to its low cost, rapid turnaround time, and availability in most clinical laboratories, CRP represents a practical biomarker candidate for use in endemic regions where melioidosis is prevalent.

Several studies have reported elevated CRP levels in patients with melioidosis, often exceeding levels observed in patients with other febrile illnesses or healthy controls. Ashdown et al. (1992) described markedly elevated CRP levels in culture-confirmed melioidosis cases, with median values exceeding 150 mg/L [8]. In a retrospective cohort study, Cheng et al. (2004; 2007) reported that patients with melioidosis had significantly higher CRP concentrations at presentation compared with patients with other infections, suggesting a potential diagnostic role [9,10]. Similarly, Natesan et al. (2017) [11] found that CRP levels were significantly higher in patients with melioidosis compared with those with other tropical infections such as leptospirosis and scrub typhus. Hui et al. (2022), in a Malaysian cohort, demonstrated that elevated CRP levels were associated with increased disease severity and worse clinical outcomes, including intensive care unit (ICU) admission [12]. Menon et al. (2021) reported that CRP values greater than 100 mg/L were significantly associated with mortality, with an odds ratio of nearly 7 for in-hospital death [13]. Zheng et al. (2023) observed that hs-CRP levels remained persistently elevated in melioidosis cases compared with healthy controls and noted a correlation between CRP values and clinical severity scores [14].

Despite these findings, the overall evidence base remains heterogeneous. Some studies, such as Chou et al. (2007), reported elevated CRP levels but lacked stratification by clinical severity, limiting their interpretability [15]. Others, including Cheng et al. (2007), presented CRP values without comparator groups [10], which hinders direct assessment of diagnostic performance. Variability in study design, sample size, assay methodology, and patient characteristics further contributes to the variability observed in CRP data across the literature. Compared with other biomarkers, such as procalcitonin or interleukin-6, which may provide greater specificity but are more costly and less widely available, CRP offers a pragmatic advantage in melioidosis-endemic regions. Its low cost, rapid turnaround time, and near-universal availability in clinical laboratories make CRP particularly suitable as a candidate biomarker for early diagnosis and risk stratification in resource-limited settings.

To better understand the role of CRP in melioidosis, we conducted a systematic review and meta-analysis of observational studies that reported CRP levels in patients with confirmed infection. This analysis sought to estimate pooled mean CRP levels and to interpret these findings in the context of typical reference ranges or, where available, data from non-melioidosis populations. We also examined how CRP levels related to markers of disease severity and clinical outcomes. By integrating data from diverse geographical and clinical settings, this study aims to assess the potential of CRP as a practical biomarker for early detection and risk stratification in melioidosis. The aims of this meta-analysis were to (1) estimate pooled CRP levels in melioidosis and explore potential diagnostic thresholds; (2) compare CRP levels in melioidosis with those in other infections or healthy controls, where available; and (3) examine correlations between CRP levels and disease severity or mortality.

## 2. Materials and Methods

### 2.1. Protocol Registration

This systematic review and meta-analysis followed the Preferred Reporting Items for Systematic Reviews and Meta-Analyses (PRISMA) guidelines. The protocol was registered prospectively with the International Prospective Register of Systematic Reviews (PROSPERO) under registration ID CRD420251069293.

### 2.2. Search Strategy

We conducted a comprehensive search of three major databases (PubMed, Embase, and Scopus) from their inception until 8 May 2025. The search terms included variations and combinations of “C-reactive protein,” “CRP,” “hs-CRP,” “melioidosis,” and “*Burkholderia pseudomallei*.” No restrictions were placed on language or publication years. Additional studies were identified through manual screening of the reference lists of included articles and relevant review papers. The details of search strategies are in Supplementary File S1.

### 2.3. Eligibility Criteria

Studies were eligible for inclusion if they met the following criteria: (1) observational design (prospective or retrospective cohort studies or case-control studies); (2) inclusion of human participants with laboratory-confirmed melioidosis; (3) quantitative measurement of CRP levels, reported as mean ± standard deviation (SD) or median with interquartile range (IQR); and (4) investigation of CRP in the context of diagnosis, severity assessment, or evaluation of clinical outcomes. Studies without outcome data were eligible if CRP measurements were collected for diagnostic or severity assessment purposes.

We excluded case reports, review articles, conference abstracts, animal studies, and studies with fewer than 10 participants. In cases of multiple publications using overlapping datasets, only the most comprehensive or recent study was included. Studies were included if they reported both laboratory confirmation of melioidosis (e.g., positive culture for *Burkholderia pseudomallei*) and quantitative CRP values. Studies reporting culture results without CRP, or CRP values without confirmed diagnosis, were excluded. To reflect the early disease phase, CRP values had to be measured at the time of clinical presentation or hospital admission; values obtained exclusively during follow-up were excluded. This approach reflects common clinical practice in endemic settings and supports the intended role of CRP as an early diagnostic biomarker.

### 2.4. Study Selection

Two reviewers (A.P. and W.K.K) independently screened all titles and abstracts based on the predefined eligibility criteria. Full-text articles were retrieved and assessed for inclusion. Any disagreements regarding study eligibility were resolved through discussion or consultation with a third reviewer (M.C.). The entire study selection process was documented in a PRISMA flow diagram.

### 2.5. Data Extraction

Two reviewers independently extracted data using a standardized form. Extracted variables included first author, year of publication, study site and country, study design, study period, total number of participants, number of melioidosis cases and controls, participant age and sex, CRP level (mean ± SD or median with IQR), method of CRP measurement, and reported clinical outcomes. In cases where data were incomplete or unclear, corresponding authors were contacted for clarification.

### 2.6. Quality Assessment

The quality of the included studies was assessed using the Newcastle–Ottawa Scale (NOS) for observational studies. NOS evaluates studies across three domains: selection of participants (maximum of 4 points), comparability of study groups (maximum of 2 points), and outcome ascertainment (maximum of 3 points). The maximum total score is 9 points. Studies were classified as high quality (7–9 points), moderate quality (5–6 points), or low quality (<5 points). Quality assessment was conducted independently by two reviewers, and discrepancies were resolved by consensus.

### 2.7. Data Synthesis and Statistical Analysis

The extracted data included sample size (*n*), mean CRP levels (mg/L), and standard deviation (SD). For studies that reported CRP levels as medians with interquartile ranges (IQRs), the mean and SD were estimated using the method proposed by Luo et al. (2018) [16]. The mean was approximated as (Q1 + Median + Q3)/3, and the SD as (Q3 − Q1)/1.35, assuming a normal distribution. These conversions enabled inclusion of studies reporting summary statistics in non-parametric form. The synthesized CRP data for all included studies are presented in Supplementary Table S1.

To ensure comparability across studies, all CRP values were standardized to milligrams per liter (mg/L). When studies reported concentrations in micrograms per milliliter (μg/mL) or international units per liter (IU/L), values were converted using the equivalences 1 μg/mL = 1 mg/L and 1 IU/L = 1 mg/L for CRP assays. Converted values are reported alongside the original units in parentheses.

Meta-analyses were performed using a random-effects model, which incorporates both within-study and between-study variability to provide more conservative pooled estimates. Between-study heterogeneity was quantified using Cochran’s Q test, the *I*^2^ statistic, and *Tau*^2^ (τ^2^). *I*^2^ values exceeding 50% were interpreted as indicating moderate to high heterogeneity, while τ^2^ provided an estimate of the actual between-study variance in CRP levels. Forest plots were generated to visually display individual study estimates and the pooled standardized mean difference (SMD) with 95% Confidence Intervals (CIs).

Exploration of potential sources of heterogeneity included assay type (standard CRP vs. hs-CRP) as an exploratory moderator. We considered performing a formal meta-regression; however, with only seven studies and limited, inconsistently reported covariates, a multivariable model was deemed statistically underpowered and potentially misleading. We therefore did not perform meta-regression and instead relied on subgroup analysis and sensitivity testing.

#### 2.7.1. Subgroup Analysis

Subgroup analyses were conducted to explore potential sources of heterogeneity by stratifying studies based on CRP assay type (standard CRP vs. hs-CRP). Differences between subgroups were formally tested using χ^2^ statistics to determine whether mean CRP levels varied significantly according to assay method under both common effect and random-effects models. Additionally, sensitivity analyses were performed by sequentially excluding studies identified as statistical outliers to examine their impact on pooled estimates and heterogeneity. Publication bias was assessed using Egger’s regression test.

#### 2.7.2. Sensitivity Analysis

To test the robustness of the findings, sensitivity analyses were conducted by sequentially removing studies identified as statistical outliers based on extreme SMD values or methodological differences. Specifically, Chou et al. (2007) [15] and Natesan et al. (2017) [11] were excluded individually and in combination. Changes in pooled CRP levels and heterogeneity were evaluated in each scenario to determine whether the overall results were disproportionately influenced by any single study.

#### 2.7.3. Publication Bias

Although the number of included studies (*n* = 7) was below the threshold for reliable funnel plot interpretation, we performed visual inspection and applied Egger’s test as an exploratory assessment. This test regresses the standard normal deviate (SMD divided by SE) against the standard error (SE). A non-significant intercept (*p* = 0.37) indicated no evidence of small-study effects or publication bias.

All analyses were conducted using R software (version 4.4.3) with the “meta” package (R Foundation for Statistical Computing, Vienna, Austria).

## 3. Results

### 3.1. Search Results

**Figure 1:**
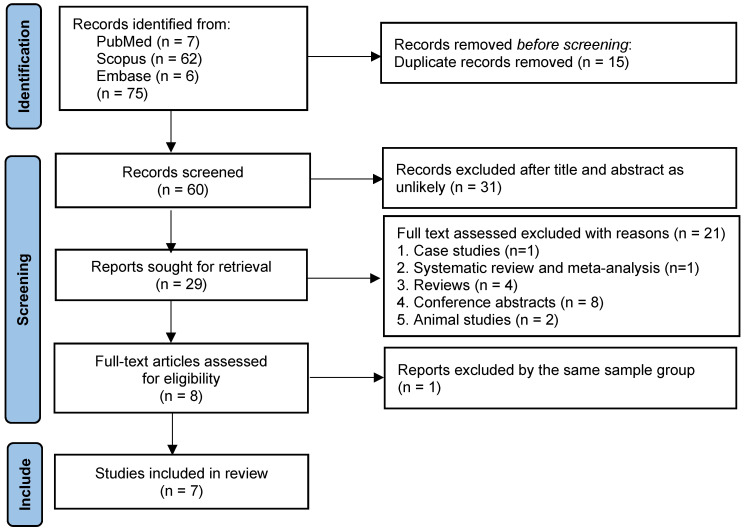
PRISMA flow diagram of study selection. The diagram details the number of records identified, screened, excluded (with reasons), and included in the meta-analysis.

The systematic search identified a total of 75 studies across three major databases: PubMed (7), Scopus (62), and Embase (6). Studies were screened according to predefined inclusion and exclusion criteria. Following screening, several studies were excluded because they involved duplicate cohort data, such as that of Cheng et al. (2007) [10], which used the same patient population as that of Cheng et al. (2004) [9]; the latter was retained for its more complete dataset. Other exclusions were due to clinically non-comparable populations, including ICU-only descriptive series or highly selected subgroups such as predominantly immunosuppressed patients, which were considered only in sensitivity analyses. Case reports, animal studies, review articles, conference abstracts, and studies with fewer than 10 participants were also excluded to maintain methodological comparability. In addition, studies with CRP values markedly different from the rest of the included literature were treated as statistical outliers and examined separately in sensitivity analyses to assess their impact on pooled estimates. These exclusions were applied to reduce bias and strengthen the robustness of the findings. The final dataset included seven observational studies, published between 1992 and 2023, conducted across diverse melioidosis-endemic regions, with sample sizes ranging from 30 to 175 participants. Further details are provided in the PRISMA flow diagram (Figure 1).

### 3.2. Characteristics of the Included Studies

The seven studies included in this meta-analysis were published between 1992 and 2023 and were conducted in melioidosis-endemic regions, including northern Australia, Taiwan, Malaysia, India, Sri Lanka, and China. Study designs comprised five retrospective cohorts, two prospective observational studies, and one descriptive case series, with sample sizes ranging from 30 to 175 patients. The studies were carried out in diverse healthcare settings, such as tertiary hospitals, intensive care units (ICUs), and regional referral centers, providing a broad context for evaluating CRP levels in melioidosis. All included studies were conducted primarily in adult populations. Only one study (Zheng et al., 2023) included a single participant aged <20 years (1/90), but no pediatric subgroup analysis was reported [14]. Therefore, the overall evidence base reflects adult patients.

Across the included studies, CRP was generally measured at or shortly after patient presentation, before laboratory confirmation of melioidosis. The interval from CRP measurement to microbiological confirmation was determined by culture turnaround times and was not explicitly reported in most studies. None of the studies used CRP values obtained substantially after initial presentation as the primary data point.

Reported CRP values varied widely both between and within studies, with ranges now provided in Table 1 alongside mean ± SD or median (IQR) values. Where comparator data were available, CRP levels in melioidosis were consistently higher than in other infections. For example, Cheng et al. (2004) reported median CRP levels of 164 mg/L in melioidosis compared with substantially lower levels in patients with other febrile illnesses [9].

Most participants were male, with proportions ranging from 67.4% (Hui et al., 2022) [12] to 87.8% (Zheng et al., 2023) [14]. Reported ages generally fell between the fifth and seventh decades of life, with mean values of 63.7 years in Chou et al. (2007) [15] and 54.5 ± 14.3 years in Hui et al. (2022) [12]. Many studies also described a high burden of comorbidities, such as diabetes mellitus, chronic kidney disease, and hazardous alcohol use—factors that can influence inflammatory markers like CRP.

CRP levels were measured using various assays. For example, Cheng et al. (2004) employed an immunoturbidimetric method [9], whereas Natesan et al. (2017) utilized ELISA [11]. Reported values included means with standard deviations or medians with interquartile ranges, Ashdown et al. (1992) found a median CRP of 154 mg/L (range 9–352 mg/L) [8], and Zheng et al. (2023) reported a mean hs-CRP of 149.57 ± 13.65 mg/L [14]. A detailed classification of assay types for each included study is provided in Table 1, along with the basis for classification (author-reported method, stated manufacturer, or inference from reported range and cutoffs). Differences in calibration, analytical range, and precision between standard CRP and hs-CRP assays are well recognized and may partly account for the variation in absolute CRP values observed. This reinforces the importance of assay harmonization in future melioidosis biomarker research.

Several individual studies also investigated the association between CRP levels and markers of disease severity. Menon et al. (2021) [13] reported that CRP levels above 100 mg/L were linked to increased mortality risk. Hui et al. (2022) [12] described higher CRP levels in patients requiring ICU admission, while Natesan et al. (2017) [11] highlighted reductions in CRP as a marker of response to treatment.

Together, these studies provide data on CRP levels in melioidosis across different populations and clinical settings. However, due to variations in study design, patient characteristics, and laboratory methods, some heterogeneity was evident in the reported CRP values.

### 3.3. Quality of Included Studies

The quality of the studies included in this meta-analysis was assessed using the Newcastle–Ottawa Scale (NOS), and the results are presented in Supplementary Table S2. Based on the NOS evaluation, the studies generally exhibited high quality, with most studies (*n* = 5) receiving a total score of 8 or 9, indicating robust methodologies for participant selection, comparability of groups, and reliable outcome measurements. These high-quality studies provide strong evidence for evaluating CRP as a diagnostic and prognostic biomarker in melioidosis. However, two studies (Ashdown et al. (1992) and Chou et al. (2007)) [8,15] were classified as moderate quality (scoring 6 points), suggesting that they had some limitations in participant selection and control of confounders.

Overall, the quality assessment highlights that the majority of the studies were well designed, and the findings regarding CRP’s role in melioidosis are based on reliable and valid methodologies.

### 3.4. Pooled CRP Levels in Melioidosis Patients

**Figure 2:**
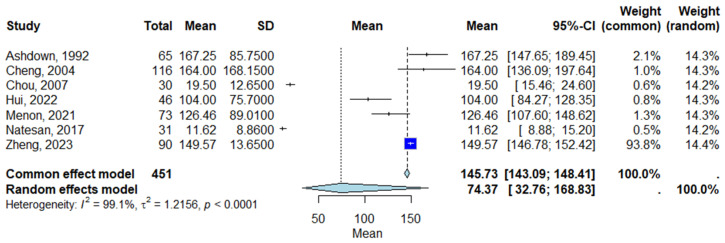
Forest plot of pooled CRP levels in patients with melioidosis [8,9,11,12,13,14,15]. Each square represents the mean CRP level for an individual study, with the size proportional to study weight. Horizontal lines indicate 95% Confidence Intervals (CI). The diamond represents the overall pooled estimate using a random-effects model. Abbreviations: CI, Confidence Interval.

Across the seven included studies, the pooled mean CRP level in patients with melioidosis was estimated to be 145.73 mg/L (95% Confidence Interval [CI]: 143.09–148.41 mg/L) under the common effect model and 74.37 mg/L (95% Confidence Interval [CI]: 32.76–168.83 mg/L) under the random-effects model, indicating substantial between-study variability. The heterogeneity for the pooled analysis was very high (*I*^2^ = 99.1%, τ^2^ = 1.2156; *p* < 0.0001), reflecting differences in study populations, methodologies, and assay types. While no formal comparison was made with healthy controls, these pooled mean CRP levels are considerably higher than typical reference values observed in healthy individuals (commonly < 10 mg/L) [17]. Figure 2 presents the forest plot of the individual study estimates and the pooled mean CRP levels under both modeling approaches.

### 3.5. Subgroup Analysis

**Figure 3:**
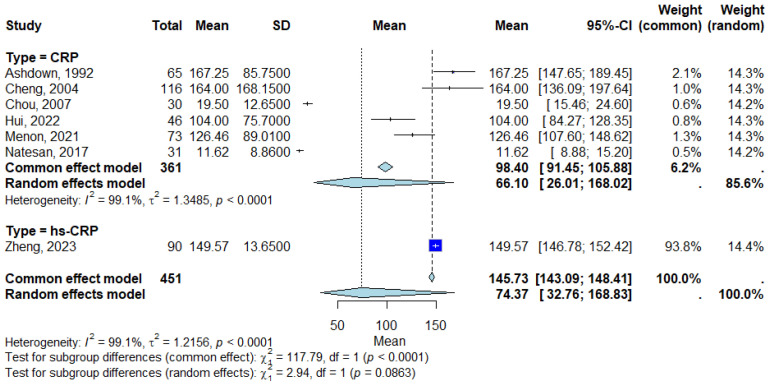
Forest plot for subgroup analysis [8,9,11,12,13,14,15]. Each square represents the mean CRP level for an individual study, with the size proportional to the study’s weight in the analysis. Horizontal lines indicate the 95% Confidence Interval (CI) for each study estimate. The diamond at the bottom of each subgroup represents the pooled mean CRP level for that subgroup, calculated using a random-effects model. Subgroups include standard CRP assays and hs-CRP assays, as defined in Table 1. The vertical line represents the null value. Abbreviations: CRP, C-reactive protein; CI, Confidence Interval; hs-CRP, high-sensitivity C-reactive protein.

Subgroup analyses were conducted to assess the impact of CRP assay type (hs-CRP vs. standard CRP), as shown in Figure 3. The studies that measured CRP showed a pooled mean CRP level of 98.40 mg/L (95% Confidence Interval [CI]: 91.45–105.88 mg/L) using the common effect model and 66.10 mg/L (95% Confidence Interval [CI]: 26.01–168.02 mg/L) using the random-effects model. The heterogeneity for the CRP subgroup was *I*^2^ = 99.1% (*τ*^2^ = 1.3485), with *p* < 0.0001 indicating high variability across studies. For studies using hs-CRP, the mean CRP level was 149.57 mg/L (95% Confidence Interval [CI]: 146.78–152.42 mg/L) in Zheng (2023), showing a significantly higher CRP value compared with standard CRP assays [14]. Figure 3 displays the forest plot of subgroup analysis, showing the pooled CRP levels for CRP and hs-CRP assays.

The test for subgroup differences revealed significant variation in CRP levels between the CRP and high-sensitivity CRP (hs-CRP) assays. Common effect model: χ^2^ = 117.79, df = 1, *p* < 0.0001, indicating a significant difference between the subgroups. Random-effects model: χ^2^ = 2.94, df = 1, *p* = 0.0863, suggesting no significant difference in variability between subgroups when accounting for heterogeneity.

### 3.6. Sensitivity Analysis

Sensitivity analyses were conducted to assess the influence of individual studies on the pooled estimates by sequentially removing potential outliers. When Chou et al. (2007), which reported the low CRP levels (mean 19.55 ± 12.65 mg/L) [15], was excluded, heterogeneity slightly decreased (*I*^2^ = 96.6%, τ^2^ = 1.0410, *p* < 0.0001). Exclusion of Natesan et al. (2017), which reported CRP in acute phase median 9.65 µg/mL (IQR 6.62–18.58) [11], which yielded relatively low values, resulted in *I*^2^ = 98.4%, τ^2^ = 0.6632, *p* < 0.0001. When both studies were removed simultaneously, between-study heterogeneity substantially declined (*I*^2^ = 79.6%, τ^2^ = 0.0281, *p* = 0.0006), while the pooled CRP levels remained significantly elevated in melioidosis patients. These results support the robustness of the findings and indicate that the meta-analysis is not unduly influenced by any single study. Full sensitivity test results are illustrated in Supplementary Figure S1.

## 4. Discussion

This systematic review and meta-analysis provide comprehensive evidence that CRP levels are substantially elevated in patients with melioidosis. By pooling data across diverse observational studies, we estimated a markedly high random-effects mean CRP level of 74.37 mg/L (95% Confidence Interval [CI]: 32.76–168.83). Although we did not perform direct statistical comparisons with healthy controls, these pooled values far exceed the commonly accepted reference range in healthy individuals (<10 mg/L) [17], supporting the clinical impression that CRP is markedly elevated in this disease. Our findings are consistent with individual studies reporting high CRP concentrations in melioidosis, often exceeding levels observed in other tropical infections such as leptospirosis, dengue, and scrub typhus [18,19,20]. Although several included studies reported CRP values for non-melioidosis infections (e.g., leptospirosis, scrub typhus, bacterial pneumonia) [18,19,20], the heterogeneity of comparator groups, inconsistency in reporting units, and variation in assay methodology precluded formal pooled analysis. This limits the ability to quantify diagnostic specificity directly. However, recent reports in severe sepsis [21,22] and bacterial pneumonia [23] have shown that CRP levels in severe bacterial infections frequently exceed 85–100 mg/L, overlapping with the high values observed in our melioidosis cohort. These findings support the role of CRP as a marker of severe bacterial infection, but its application in melioidosis should be integrated with the clinical and epidemiological context.

As part of the innate immune response, CRP binds to phosphocholine expressed on the surface of pathogens and damaged host cells, promoting opsonization and complement activation [24]. This biological mechanism explains why CRP levels can rise dramatically, often 100-fold or more, during acute bacterial infections [25], including melioidosis caused by *B. pseudomallei* [8,9]. The prompt and robust elevation of CRP in systemic bacterial infections makes it a valuable early indicator of inflammation and sepsis. In comparison, biomarkers such as procalcitonin or IL-6 may offer greater specificity but are less accessible and more costly, particularly in endemic regions. CRP therefore remains the more pragmatic biomarker for melioidosis due to its wide availability, low cost, and rapid turnaround time [7].

The utility of CRP as a diagnostic and prognostic biomarker is well recognized in a broad range of infectious diseases. Elevated CRP levels are routinely used to differentiate bacteria from viral infections [18,19,20,23], to support the diagnosis of community-acquired pneumonia, and to guide antibiotic stewardship. In tropical medicine, CRP has been explored as a means to distinguish severe malaria from bacterial sepsis [26] and to differentiate dengue from bacterial coinfections [19]. Our findings that CRP levels are markedly elevated in melioidosis compared with typical healthy reference ranges, and even relative to other febrile illnesses, further emphasize its diagnostic potential in endemic regions where the differential diagnosis is broad and often uncertain.

Our subgroup analysis revealed higher pooled CRP values in studies employing hs-CRP assays, suggesting that assay type may contribute to variability across studies. This is consistent with observations in other infectious diseases where hs-CRP provides enhanced sensitivity for detecting inflammatory states [27]. Standard CRP assays are primarily designed to measure higher concentration ranges (5–500 mg/L), making them ideal for acute infections, whereas hs-CRP assays are optimized for much lower concentrations (0.1–10 mg/L) to detect subtle chronic inflammation, such as in cardiovascular risk assessment [27]. Despite this, hs-CRP assays can still quantify very high CRP levels, as seen in our included study by Zheng et al. (2023), which reported mean values exceeding 140 mg/L [14]. Differences in calibration and analytical precision at various ranges may therefore partially explain why pooled CRP levels appeared higher in hs-CRP studies [28]. However, the test for subgroup differences using the random-effects model did not reach statistical significance, indicating that while assay type may influence absolute values, it does not entirely account for the substantial between-study heterogeneity observed in this meta-analysis.

The very high between-study heterogeneity (*I*^2^ = 99.1%) likely reflects differences in patient characteristics, severity mix (ICU vs. non-ICU), geography, and assay methodology. Under such circumstances, pooled means should be interpreted as directional evidence of marked systemic inflammation rather than precise diagnostic thresholds. Although a formal meta-regression was considered, the small sample size (*n* = 7) and incomplete reporting of covariates precluded a robust analysis. For the same reasons, additional univariable explorations (e.g., by year or geography) were not performed, as they would have been underpowered and potentially misleading. We therefore emphasize that the pooled mean CRP values should be regarded as descriptive and hypothesis-generating only. This limitation highlights the need for larger standardized prospective studies to better evaluate sources of heterogeneity. Our approach, combining exploratory subgrouping by assay type with sensitivity analyses, provided a practical means of assessing the robustness of our findings while minimizing the risk of spurious conclusions. Importantly, these pooled values should not be interpreted as clinically actionable thresholds but rather as a summary of the current heterogeneous evidence.

Importantly, sensitivity analyses demonstrated the robustness of our findings. Sequential exclusion of studies reporting notably lower CRP levels, such as Chou et al. (2007) [15] and Natesan et al. (2017) [11], reduced heterogeneity from 99.1% to 79.6% without altering the conclusion that CRP is significantly elevated in melioidosis. Similarly, exclusion of moderate-quality or methodologically atypical studies, including Ashdown (1992) [8] and Chou et al. (2007) [15], yielded consistent pooled estimates. These results indicate that while such studies contribute to between-study variability, they do not materially affect the direction or magnitude of the association. Overall, removing methodologically or clinically non-comparable studies, as well as statistical outliers, strengthened the internal validity of our pooled estimates and confirmed that the elevated CRP levels observed are robust across diverse study settings and populations.

The clinical implications of these results are considerable, particularly in endemic areas where rapid, affordable, and widely available biomarkers are essential for timely diagnosis and risk stratification. Given the high mortality associated with melioidosis, early identification of severe cases using CRP could facilitate prompt initiation of appropriate antibiotic therapy and supportive care. Several included studies also indicated that higher CRP levels correlate with worse outcomes, including increased mortality and ICU admission. Based on prior studies linking elevated CRP with poor outcomes, we propose a threshold of >100 mg/L as a research-grade cutoff. This should be regarded as hypothesis-generating only and cannot be applied clinically without prospective validation.

Despite these strengths, our meta-analysis has several limitations. First, the analysis included a relatively small number of studies (*n* = 7), which limits the power of subgroup analyses and the reliability of publication bias assessments. Although Egger’s test indicated no evidence of publication bias (*p* = 0.37), the small number of studies included (*n* = 7) means that the absence of bias cannot be confidently concluded. In addition, CRP levels are known to change rapidly with disease progression and treatment response, yet most studies reported only a single time-point measurement. This limits the ability to assess dynamic trends in CRP and their association with outcomes. Additionally, substantial heterogeneity persisted even after sensitivity analyses, likely due to differences in study design, population characteristics, baseline comorbidities (such as diabetes and chronic kidney disease), and laboratory methods. The studies included in this meta-analysis employed diverse CRP assays, including standard immunoturbidimetric tests, enzyme-linked immunosorbent assays (ELISAs), and hs-CRP methods, which may differ in analytical sensitivity, calibration, and precision at high concentration ranges, contributing further to variability in reported CRP levels. Another important limitation is that CRP is a non-specific acute-phase reactant that can be elevated in a wide range of infectious and inflammatory conditions; thus, elevated CRP alone cannot definitively distinguish melioidosis from other severe bacterial or inflammatory diseases. Finally, most included studies were observational in nature, introducing potential residual confounding that may affect the associations between CRP levels and clinical outcomes, even though the majority were assessed as high quality using the Newcastle–Ottawa Scale.

Future research should aim to standardize CRP assay methodologies and investigate threshold values that optimize diagnostic and prognostic performance specifically in melioidosis. Prospective studies are also needed to evaluate the integration of CRP with other clinical and laboratory parameters to develop robust predictive models for disease severity and outcomes.

## 5. Conclusions

C-reactive protein (CRP) is a widely available and low-cost biomarker that is consistently elevated in patients with melioidosis. While our findings support its potential role in early diagnosis and severity assessment, the high heterogeneity among studies limits the precision and generalizability of pooled estimates. CRP should therefore be integrated with other biomarkers (e.g., procalcitonin) and clinical scoring systems to enhance diagnostic and prognostic accuracy, rather than being used in isolation. Future prospective studies are needed to establish and validate clinically relevant CRP thresholds for melioidosis and to assess their performance when combined with other clinical and laboratory parameters in prediction models.

## Figures and Tables

**Table 1:** Characteristics of the 7 included studies.

First Author (Ref.)	Year of Publication	Study Site	Study Design	Year Conducted	Participant Description	Number of Cases	% Male	Age (Mean ± SD or Range) Year	CRP Level (Mean ± SD or Median (IQR))	CRP Type	Notes: Basis for Classification and Others
Ashdown [8]	1992	Darwin, Australia	Prospective observational	1989–1990	Patients admitted with culture-confirmed melioidosis	65	NR	NR	Median = 154.0 mg/L (range 9.0–352.0)	Standard CRP	Inferred from era and high-range reporting. Author reported Abbott^®^ TDx^®^ automate
Cheng [9]	2004	Darwin, Australia	Retrospective cohort	1995–2002	Patients with confirmed melioidosis and other febrile illnesses	116	NR	NR	Median 164.0 mg/L(IQR) of 59.0–286.0 mg/L	Standard CRP	Author-reported immunoturbidimetric assay
Chou [15]	2007	Taiwan	Case series	2002–2006	Melioidosis cases from ICU	30	80%	Mean 63.7 years	Mean ± SD = 19.55 ± 12.65 mg/L	Standard CRP	Inferred from clinical context and range. Severe cases, small sample
Hui [12]	2022	Malaysia	Retrospective cohort	2015–2019	Culture-confirmed melioidosis patients	46	67.4%	54.5 ± 14.3 years	Median = 104.0 mg/L (IQR 52.9–155.1)	Standard CRP	Assumed (no hs-CRP label; high median values). Reported CRP trends with severity
Menon [13]	2021	Kochi, India	Retrospective cohort	2011–2020	Hospitalized melioidosis cases	73	NR	NR	^#^ Mean ± SD = 126.46 ± 89.01 mg/L	Standard CRP	Assumed from clinical context; manufacturer not specified. Logistic regression showed CRP as mortality predictor
Natesan [11]	2017	Sri Lanka	Prospective observational	2014–2015	Confirmed case of melioidosis with diabetes (74%), alcoholism (10%), kidney disease (10%), and other comorbidities (20%). Blood serum samples were collected during the acute and eradication phases of the antibiotic treatment	31	74%	Mean 50 (range 32–74)	^##^ Acute phase median = 9.7 mg/L (IQR 6.6–18.6); eradication median = 2.9 mg/L (IQR 6.7–8.6)	Standard CRP	Assumed from clinical context. Author-reported “enzyme-linked immunosorbent assay (ELISA)”. Also evaluated calprotectin
Zheng [14]	2023	Hainan, China	Retrospective cohort	2010–2020	Multicenter hospitalized cases	90	87.8%	Range 0–81	hs-CRP: mean ± SD = 149.57 ± 13.65 mg/L	hs-CRP	Author-reported “hs-CRP”. Explored rainfall correlations. Procalcitonin level of 1.31 (0.39, 6.21) ng/mL

NR: not reported in the original study; SD: standard deviation; CRP: C-reactive protein; hs-CRP: high-sensitivity C-reactive protein; ICU: intensive care unit. All CRP values were standardized to mg/L. Original units reported as IU/L (^#^) or µg/mL (^##^). Full details of conversions from median (IQR) to mean ± SD are presented in Supplementary Table S1.

## Data Availability

Not applicable.

## Supplementary Materials

The following supporting information can be downloaded at https://www.mdpi.com/article/10.3390/life15091360/s1: Supplementary File S1: Search strategies; Supplementary Table S1: The synthesized CRP data; Supplementary Table S2: The quality assessment of the studies; Supplementary Figure S1: Forest plots of sensitivity analyses.

## Abbreviations

The following abbreviations are used in this manuscript:

CRP	C-reactive protein
ELISA	enzyme-linked immunosorbent assay
hs-CRP	High-sensitivity C-reactive protein
ICU	intensive care unit
IL-6	interleukin-6
IU	international unit
mg	milligram
mL	milliliter
NOS	Newcastle–Ottawa Scale
µg	microgram
