# Supplementary material for: C-Reactive Protein for Early Diagnosis and Severity Monitoring in Melioidosis: A Systematic Review and Meta-Analysis
Source: Life (Basel). 2025 Aug 27;15(9):1360. doi: 10.3390/life15091360 (PMC12471701; doi:10.3390/life15091360)
Supplement: Supplementary file 1 [file life-15-01360-s001.zip › Supplementary Figure S1_Sensitivity test.pdf]

A.

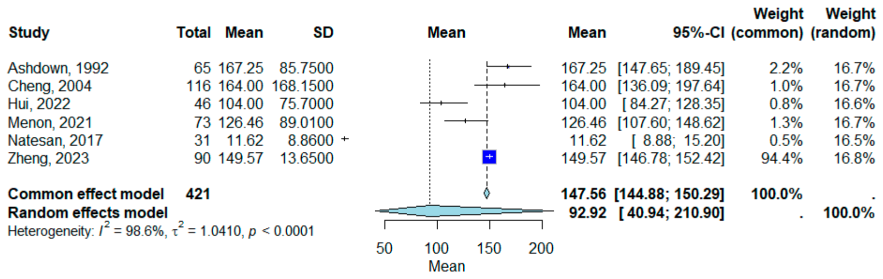

B.

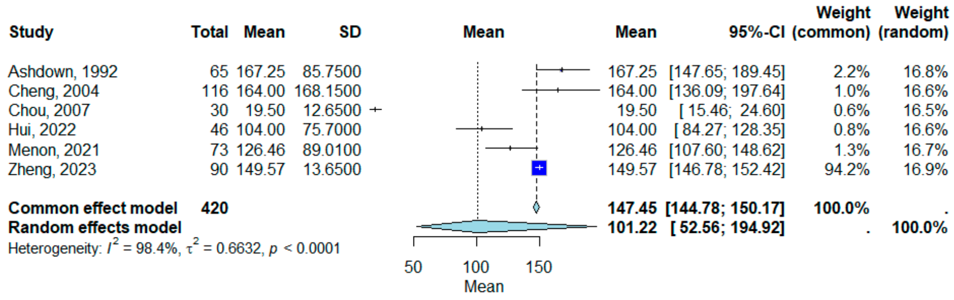

C.

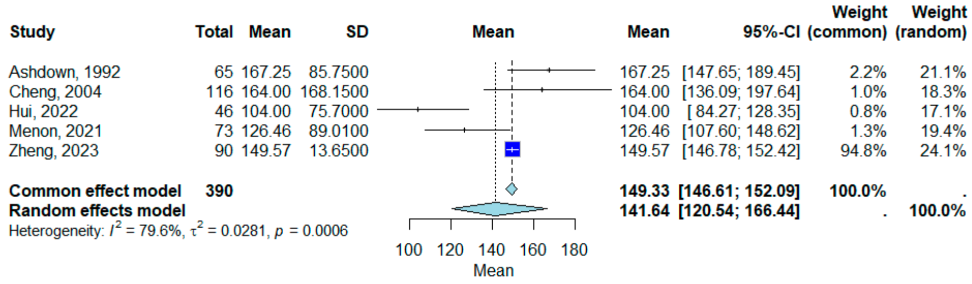

Supplementary Figure S1. Forest plots of sensitivity analyses evaluating the impact of outlier studies on the pooled CRP levels in melioidosis. Panel A shows the results after excluding Chou et al. (2007). Panel B displays the analysis excluding Natesan et al. (2017). Panel C presents the results when both studies were excluded simultaneously.
