# Supplementary material for: C-Reactive Protein for Early Diagnosis and Severity Monitoring in Melioidosis: A Systematic Review and Meta-Analysis
Source: Life (Basel). 2025 Aug 27;15(9):1360. doi: 10.3390/life15091360 (PMC12471701; doi:10.3390/life15091360)
Supplement: Supplementary file 1 [file life-15-01360-s001.zip › Supplementary File S1_Search stratergy.pdf]

Supplementary File S1. Search strategies for 3 databases. The inception date is Jun 5th, 2025.

PubMed

"C-reactive protein" OR "C-reactive protein" OR "CRP" OR "hs-CRP" AND "Burkholderia pseudomallei" OR "melioidosis" OR "B. pseudomallei"

Filter:

Filters applied: Full text, Classical Article, Clinical Study, Comparative Study, Observational Study, Randomized Controlled Trial.

Result: 189

SCOPUS

"C-reactive protein" OR "C-reactive protein" OR "CRP" OR "hs-CRP" AND "Burkholderia pseudomallei" OR "melioidosis" OR "B. pseudomallei" AND (LIMIT-TO (DOCTYPE , "ar")) AND (LIMIT-TO (PUBSTAGE , "final")) AND (EXCLUDE (EXACTKEYWORD , "Case Report") OR EXCLUDE (EXACTKEYWORD , "Nonhuman") OR EXCLUDE (EXACTKEYWORD , "Mouse") OR EXCLUDE (EXACTKEYWORD , "Animal Model") OR EXCLUDE (EXACTKEYWORD , "Mice") OR EXCLUDE (EXACTKEYWORD , "Animal Experiment") OR EXCLUDE (EXACTKEYWORD , "Animals")) AND (LIMIT-TO (SRCTYPE , "j")) AND (LIMIT-TO (LANGUAGE , "English"))

Result: 212

Embase

No	Query Results	Results
#3	#1 AND #2	213
#2	.'burkholderia pseudomallei' OR 'melioidosis' OR 'b. pseudomallei'	6,348
#1	'c-reactive protein' OR 'crp' OR 'hs-crp'	372,567
