# Supplementary material for: C-Reactive Protein for Early Diagnosis and Severity Monitoring in Melioidosis: A Systematic Review and Meta-Analysis
Source: Life (Basel). 2025 Aug 27;15(9):1360. doi: 10.3390/life15091360 (PMC12471701; doi:10.3390/life15091360)
Supplement: Supplementary file 1 [file life-15-01360-s001.zip › Supplementary Table S1_Synthetic CRP data.pdf]

Supplementary Table S1. Synthetic CRP data of the included studies.

Author	N	Mean	SD	Type
Ashdown, 1992	65	167.25	85.75	CRP
Cheng, 2004	116	164	168.15	CRP
Chou, 2007	30	19.5	12.65	CRP
Hui, 2022	46	104	75.7	CRP
Menon, 2021	73	126.46	89.01	CRP
Natesan, 2017	31	11.62	8.86	CRP
Zheng, 2023	90	149.57	13.65	hs-CRP
