# Supplementary material for: C-Reactive Protein for Early Diagnosis and Severity Monitoring in Melioidosis: A Systematic Review and Meta-Analysis
Source: Life (Basel). 2025 Aug 27;15(9):1360. doi: 10.3390/life15091360 (PMC12471701; doi:10.3390/life15091360)
Supplement: Supplementary file 1 [file life-15-01360-s001.zip › Supplementary Table S2_Quality Assessment.pdf]

Supplementary Table S2. The Quality Assessment of the studies included in the meta-analysis was conducted using the Newcastle-Ottawa Scale (NOS).

Study	Selection of Participants	Comparability of Study Groups	Outcome Ascertainment	Total Score (out of 9)	Quality Classification
**Ashdown et al. (1992)**	3/4	1/2	2/3	6	Moderate
**Cheng et al. (2004)**	4/4	1/2	3/3	8	High
**Chou et al. (2007)**	3/4	1/2	2/3	6	Moderate
**Hui et al. (2022)**	4/4	2/2	3/3	9	High
**Menon et al. (2021)**	3/4	2/2	3/3	8	High
**Natesan et al. (2017)**	3/4	2/2	3/3	8	High
**Zheng et al. (2023)**	4/4	2/2	3/3	9	High
